# Insights into γ-valerolactone-mediated lignin recovery and its adsorption performance for methylene blue

**DOI:** 10.1039/d6ra03476d

**Published:** 2026-07-06

**Authors:** Lingmei Yang, Longlong Zhao, An Wei, Guoling Zhang, Lianhua Li, Bing Han

**Affiliations:** a School of Shipping and Maritime Studies, Guangzhou Maritime University Guangzhou 510725 China guoling0620@163.com; b Key Laboratory of Renewable Energy, Guangzhou Institute of Energy Conversion, Chinese Academy of Sciences Guangzhou 510640 China lilh@ms.giec.ac.cn

## Abstract

The recovery of lignin from sweet potato vines *via* γ-valerolactone (GVL)-mediated processes with different GVL addition sequences during pretreatment and acid precipitation sequences was systematically investigated. The GVL dosing sequence was identified as the core factor determining lignin recovery efficiency and purity. For Process I (GVL added during the pretreatment stage, denoted as GVL/NaOH-LP), the maximum lignin recovery yield of 0.38 ± 0.02 g was achieved, albeit with the lowest purity of 47.79% ± 1.44%. By comparison, Process III (GVL added prior to acid precipitation, denoted as NaOH/GVL-LP) produced lignin with a significantly improved purity of 82.48% ± 0.001%, but the recovered mass was only 0.19 ± 0.002 g, indicating that process optimization requires a balance between recovery yield and product purity. The adsorption behavior of methylene blue (MB) on GVL/NaOH-LP was fitted well by the Langmuir isotherm model and pseudo-second-order kinetic model (*R*^2^ = 0.8616), with a maximum adsorption capacity of 38.75 mg g^−1^. Characterization confirmed that MB adsorption on lignin particles was driven by a synergistic mechanism involving π–π stacking interactions, functional groups, and a mesopore-facilitated adsorption process.

## Abstract 2

The recovery of lignin from sweet potato vines *via* γ-valerolactone (GVL)-mediated processes with different GVL addition sequences during pretreatment and acid precipitation sequences was systematically investigated.

## Graphical abstract

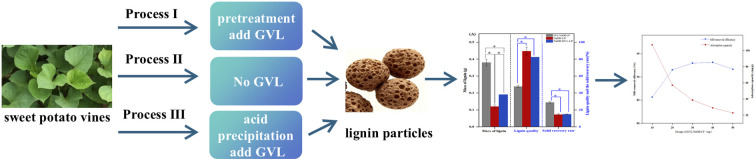

## 1 Introduction

As one of the principal components of plant cell walls together with cellulose and hemicellulose, lignin is the most abundant naturally-exhibiting aromatic polymer, with a proportion of 15–30% of the total biomass.^1–3^ The combinatorial nature of lignin's polymerization with the three typical lignin monomers (guaiacyl, syringyl and *p*-hydroxyphenyl) results in various reactive groups on the final polymer, namely free aliphatic and phenolic hydroxyl groups, as well as methoxy and carboxylic groups.^4^ The structural and chemical features of lignin make it a promising raw material for producing platform chemicals, advanced functional materials and efficient environmental remediation agents.^5^ Therefore, the extraction and separation of lignin stands out as the most critical procedure in the whole process. Currently, physical, chemical, and biological strategies are adopted to separate lignin.^6^ Among them, chemical approaches are most commonly used, which involve acid hydrolysis, alkaline dissolution (NaOH, calcium hydroxide, and potassium hydroxide), organic solvents (low-molecular-weight aliphatic alcohols, organic acids, and mixed solvents), and ionic liquids.^7,8^ For example, bagasse treated with NaOH^9^ at 35 °C for 8 days and subsequent acid precipitation achieved a lignin yield of 88.6%. Similarly, the pretreatment of bamboo with phenol-promoted guanidine hydrochloride/lactic acid deep eutectic solvent resulted in a lignin yield of 100.3%.^10^ Accordingly, chemical approaches for targeted separation and efficient recovery of lignin hold considerable scientific and practical significance.

γ-Valerolactone (GVL) is a renewable bio-based solvent and exhibits unique advantages in biomass pretreatment and lignin valorization owing to its outstanding lignin solvation capacity.^4^ Previous investigation focusing on two-step hydrolytic separation of poplar wood using GVL indicated that the recovered lignin features well-preserved β-ether content (31.9%) and higher yields (56.5%), showing potential for continuous-flow application.^11^ Further research on cotton stalk pretreatment confirms the GVL-assisted deep eutectic solvent system could boost lignin removal, achieving a 96% delignification ratio under mild conditions (90 °C, 30 min, 1 : 20).^12^ Furthermore, GVL-mediated extraction is performed within a temperature range of 140 °C to 200 °C, which effectively mitigates condensation and structural degradation caused by overheating.^13^ Moreover, GVL can also be efficiently recovered *via* distillation, making it well aligned with green chemistry principles. Thus, GVL has emerged as a promising medium for stripping lignin from various agricultural residues, achieving lignin recovery yields higher than 70%.^14,15^ However, the process parameters governing lignin recovery efficiency require systematic investigation to establish the GVL-mediated lignin separation systems.

Recovered lignin exhibits considerable adsorption capacity toward cationic dyes such as methylene blue (MB) from aqueous solution, owing to its aromatic backbone and abundant surface functional groups.^16^ As a typical cationic dye widely applied in the textile, printing, and dyeing industries, MB features high chemical stability that renders its biodegradation extremely challenging. Therefore, the discharge of MB into water bodies poses severe threats to ecological safety.^17^ The aerogel fabricated from holocellulose and lignin-based materials achieves superior adsorption performance toward MB, with the maximum adsorption capacity reaching 18.86 mg g^−1^ and the corresponding removal rate hitting 95.8%.^18^ Benefiting from the structural regulation by lignin introduction, MB adsorption is improved *via* synergistic effects including π–π stacking, electrostatic interaction, hydrogen bonding and physical adsorption.^19,20^ Despite significant advances in lignin-based adsorbent materials, the adsorption behavior of the recovered lignin toward MB remains insufficiently explored.

Considerably, this study utilizes sweet potato vines as the raw material to investigate how GVL-mediated pretreatment and acidic precipitation processes influence lignin recovery. To achieve this research objective, three typical processes with distinct GVL addition timings (during the pretreatment stage or prior to acidic precipitation) were established. On this basis, the study explores the effects of GVL addition timing on the recovery efficiency. To further expand its application, MB was selected as the target pollutant to evaluate the adsorption performance of lignin particles derived from the aforementioned processes. The adsorption behavior was analyzed by combining dynamic adsorption characteristics with isothermal adsorption data. Integrated results derived from lignin recovery experiments and adsorption performance tests provide theoretical fundamentals to guide the controllable recovery of lignin and promote its downstream industrial exploitation.

## 2 Experimental materials and methods

### 2.1 Raw materials

Sweet potato vines were manually cut into segments of 2–3 cm in length. The chopped material was dried, ground, and sieved through a 60–100 mesh sieve, and stored for subsequent use. The contents of cellulose, hemicellulose and lignin in the raw material were 27.68 ± 0.45%, 10.24 ± 1.05%, and 17.13 ± 0.87% on a dry weight basis, respectively.

Sodium hydroxide, GVL, and MB were purchased from Shanghai MacLean Biochemical Technology Co., Ltd., while concentrated sulfuric acid was obtained from Sinopharm Group Co., Ltd.

### 2.2 Procedures

#### 2.2.1 Recovery procedures of lignin particles

**Fig. 1:**
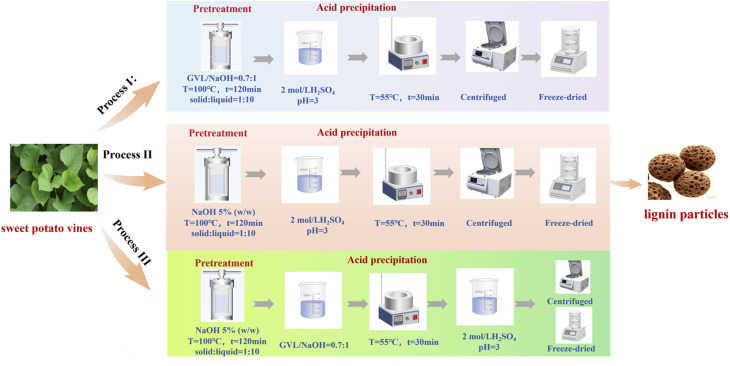
Schematic illustration of lignin particle formation *via* time-varied GVL addition protocols.

Lignin was isolated from sweet potato vines through a combined route consisting of GVL-assisted alkaline pretreatment and subsequent acid precipitation. The overall lignin extraction workflow consisted of three successive steps. Initially at the pretreatment stage, raw material was treated with 5 wt% NaOH solution at 100 °C for 120 min under a solid-to-liquid ratio of 1 : 10 (g : mL). Upon finishing the pretreatment process, the collected supernatant was further processed by acid precipitation procedure. Specifically, 2 mol L^−1^ H_2_SO_4_ was used to regulate the solution pH to 3.0, and the resulting mixture was subjected to continuously shaking at 55 °C for 30 min. After acid precipitation, the sample was centrifuged at 8000 rpm for 5 min and subsequently filtered. The recovered solid was repeatedly rinsed with deionized water to neutral pH, and further freeze-dried to obtain lignin particles. To investigate how GVL affects lignin recovery, three distinct experiments were performed to compare pretreatment performance and acid-precipitated lignin yields (Fig. 1). Among them, Process I adopted a GVL-assisted NaOH pretreatment system with a fixed GVL : NaOH molar ratio of 0.7 : 1. Process II was carried out merely on NaOH solution without GVL supplement thorough the whole process. Differing from the above two experiments, Process III performed NaOH pretreatment first, and GVL was supplemented to the collected liquid fraction to attain a 0.7 : 1 GVL : NaOH molar ratio, corresponding to an overall GVL dosage of 2.1875 g. The resulting lignin particles from Process I, II, and III were correspondingly named GVL/NaOH-LP, NaOH-LP, and NaOH/GVL-LP, respectively. All data are presented as mean values from three independent replicate tests, with error bars representing standard deviations.

#### 2.2.2 Adsorption experiment of methylene blue on lignin particles

Experiments were conducted in Erlenmeyer flasks filled with GVL/NaOH-LP and 50 mg L^−1^ methylene blue (MB) solution, then placed on a constant-temperature shaker. The mixtures were shaken continuously at 150 rpm for 4 hours to reach adsorption equilibrium. To evaluate the adsorption capacity, varying dosages of GVL/NaOH-LP (10, 20, 30, 40, and 50 mg) were added into 25 mL MB solutions to investigate their adsorption behaviors. The absorbance of the resulting solution was measured at 664 nm using an ultraviolet-visible (UV-Vis) spectrophotometer (Lambda 750; PerkinElmer, USA). The MB removal rate (*R*) and adsorption capacity (*q*_s_) were calculated using eqn (1) and (2).^21^1
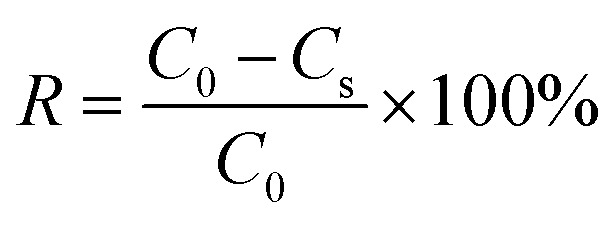
2
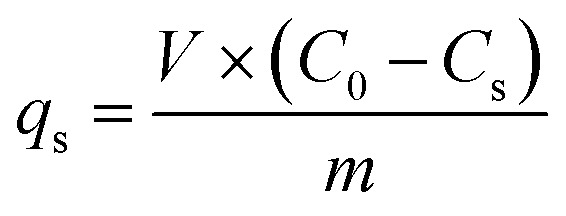
where *q*_s_—adsorption capacity at equilibrium time (mg g^−1^); *C*_0_—initial concentration of the MB solution (mg L^−1^); *C*_s_—equilibrium concentration of the MB solution (mg L^−1^); *V*—volume of MB solution (L); *m*—the quality of the GVL/NaOH-LP (mg); *R*—MB removal rate (%).

#### 2.2.3 Adsorption kinetics studies

To explore the interaction mechanisms in the adsorption process, pseudo-first-order, pseudo-second-order and intraparticle diffusion models were adopted to fit the kinetic data of MB removal by GVL/NaOH-LP (Table 1). GVL/NaOH-LP (30 mg) was mixed with 25 mL MB solution (50 mg L^−1^) and shaken continuously at 30 °C for 240 min. Supernatants were sampled at 5, 10, 15, 20, 40, 60, 80, 120, 160, 200, and 240 min. A 0.45 µm polyethersulfone membrane was used for filtration before measuring MB concentration.

**Table 1:** Models used for kinetic analysis^a^

Model	Formula
Pseudo-first-order kinetic model	*q*_*t*_ = *q*_e_(1 − e^−*k*_1_*t*^)
Pseudo-second-order kinetic model	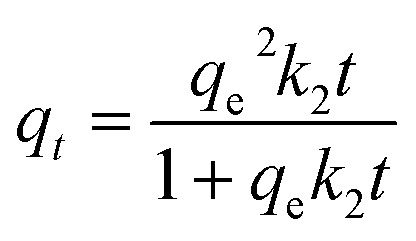
Intraparticle diffusion model	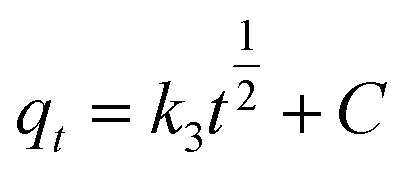

a *q*_*t*_—adsorption amounts of GVL/NaOH-LP for MB at time *t* (mg g^−1^); *q*_e_—adsorption amounts of GVL/NaOH-LP for MB at equilibrium *t* (mg g^−1^); *k*_1_— adsorption rate constant of pseudo-first-order kinetic model (min^−1^); *k*_2_—adsorption rate constant of pseudo-second-order kinetic model (g mg^−1^ min^−1^); *k*_3_—adsorption rate constant of intraparticle diffusion model (mg/(g min ^1/2^)); *t* —the contact time (min); *C*—a constant related to the intraparticle diffusion model (m).

#### 2.2.4 Adsorption isotherm studies

GVL/NaOH-LP (30 mg) was added to 25 mL of MB solutions with varying initial concentrations (10, 20, 30, 40, 50, 60, 70, 80, 90, and 100 mg L^−1^), followed by filtration through a 0.45 µm polyethersulfone filter after being shaken at 30 °C and 150 rpm for 240 min. To investigate the quantitative relationship between the adsorption capacity of lignin particles for MB and the initial concentration of MB, the adsorption data were analyzed using the Langmuir and Freundlich isotherm models (Table 2).

**Table 2:** Models used for adsorption isothermal analysis^a^

Model	Formula
Langmuir model	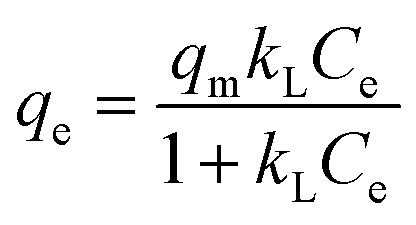
	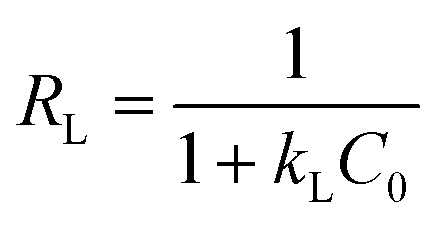
Freundlich model	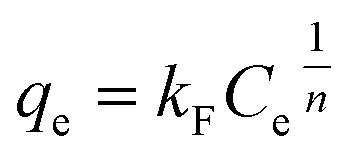

a *q*_e_—adsorption amounts of GVL/NaOH-LP for MB at equilibrium *t* (mg g^−1^); *q*_m_—saturated adsorption capacity of lignin particles for MB (mg g^−1^); *k*_L_—equilibrium constant of the Langmuir model (mg L^−1^); *R*_L_—equilibrium parameter; *C*_0_—initial concentration of MB solution (mg L^−1^); *k*_F_—Freundlich constant (mg g^−1^ (L mg^−1^)^1/*n*^); *n*—intensity of adsorption.

### 2.3 Methods for analysis and data processing

#### 2.3.1 Analysis methods

The contents of glucan (representing cellulose), xylan (representing hemicellulose), and lignin were determined according to the standard assay method for biomass analysis issued by the US National Renewable Energy Laboratory (LAP, NREL). Namely, the concentrations were measured using a Waters 2698 high-performance liquid chromatography (HPLC) system equipped with a Shodex column (SH1011). The column was maintained at a temperature of 50 °C, and dilute sulfuric acid (0.005 mM H_2_SO_4_) served as the mobile phase at a flow rate of 0.5 mL min^−1^.

The purity of the recovered lignin was calculated by combining the contents of acid-insoluble lignin and acid-soluble lignin, which were quantified *via* a two-step acid hydrolysis method integrating gravimetric and chromatographic determinations.^22^

The functional groups of the samples were analyzed by Fourier-transform infrared spectroscopy (FTIR, TENSOR27). Briefly, each sample (1 mg) was mixed with potassium bromide (150 mg) and pressed into a transparent pellet at a pressure of 10 MPa. The spectra of the pellets were acquired at a maximum resolution of 4 cm^−1^ over the scanning range of 4000–400 cm^−1^.

The surface morphology was examined using a Hitachi S-4800 field-emission scanning electron microscope (SEM) at an accelerating voltage of 5.0 kV. To minimize charging effects, the dried powder samples were mounted on conductive carbon tape attached to aluminum stubs and sputter-coated with a thin gold film (approximately 8 nm) using a Hitachi E-1045 ion sputter coater (15 mA, 60 s). Subsequently, the specimens were transferred into the SEM chamber for observation.

The samples were analyzed for their carbon (C), hydrogen (H), and nitrogen (N) concentrations through an elemental analyzer (Vario ELcube), whereas the oxygen (O) level was determined by applying the difference method.^23^ The adsorption–desorption isotherms, specific surface area, and mesopore size distribution of the samples were collected in a fully automatic physical adsorption instrument (SI-MP-10).

#### 2.3.2 Data processing methods

Statistical analyses were performed to compare differences across groups. A *P* value less than 0.05 was regarded as the threshold for statistical significance.

## 3 Results and discussion

### 3.1 Effect of pretreatment conditions on components

**Fig. 2:**
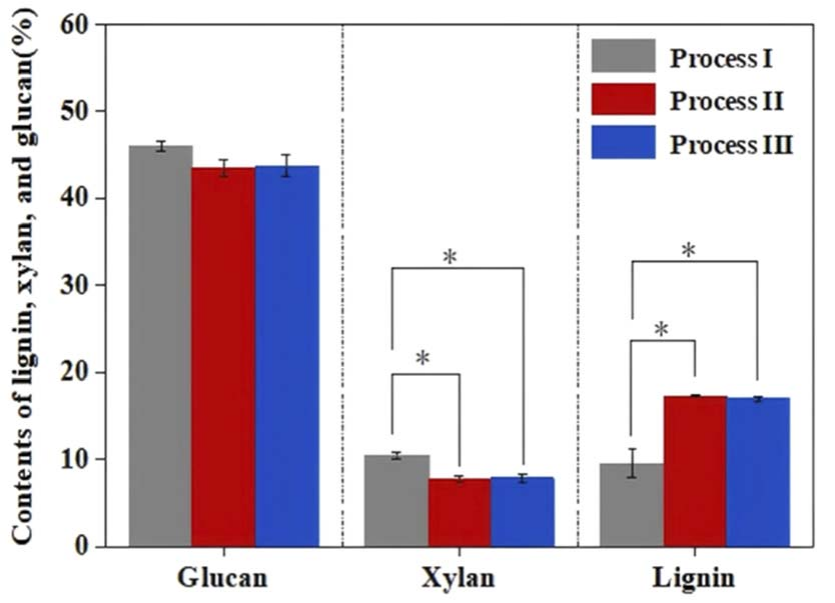
Changes in contents of three elements in the sample after pretreatment.

Experimental results indicated that adding GVL in the pretreatment process exerted a significant effect on lignin, xylan and glucan contents (Fig. 2). With respect to glucan, its content remained relatively stable, ranging from 43.51% ± 1.01% to 46.01% ± 0.51% for all the samples. When the GVL : NaOH molar ratio shifted from that of GVL/NaOH-LP (Process I) to NaOH-LP (Process II) and NaOH/GVL-LP (Process III), the xylan content dropped by 25–26%. This reveals that GVL addition inhibits xylan degradation during alkaline pretreatment.^23^ Meanwhile, compared with GVL/NaOH-LP, the lignin contents of NaOH-LP and NaOH/GVL-LP increased 78–82%. This phenomenon could be attributed to: (1) Higher temperatures promoted lignin condensation reactions, leading to the formation of lignin-like polyphenolic structures and a subsequent increase in the lignin content of pretreated samples;^24^ (2) the lignin removal reached a plateau at this temperature, which is consistent with the findings of Shinde *et al.*,^25^ who found the maximum delignification rate occurred at 120 °C and served as the saturation point that when raw materials were pretreated with GVL–water system at 100 °C, 110 °C and 120 °C.

### 3.2 Lignin recovery under different conditions

**Fig. 3:**
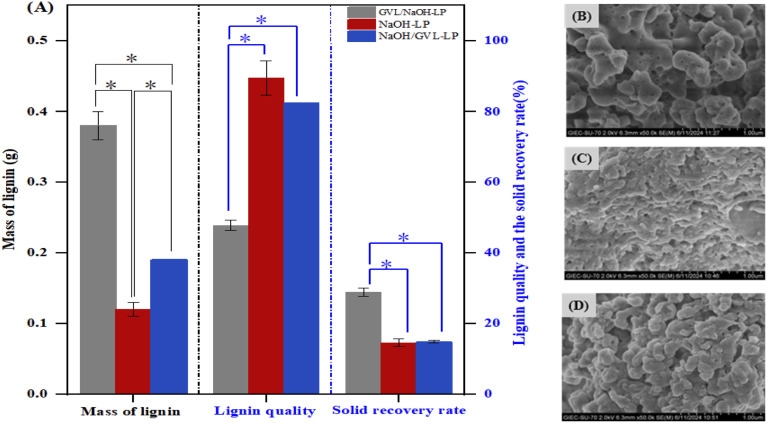
Solid recovery rate after pretreatment and properties of lignin particles obtained through acidic precipitation. (A) Solid recovery rate and properties of lignin particles; (B) SEM image of GVL/NaOH-LP; (C) SEM image of NaOH-LP; (D) SEM image of NaOH/GVL-LP.

Lignin recovered from the acidic precipitation process varied significantly with different pretreatment conditions, exhibiting an inverse trend in terms of quality and mass with considerable differences in particle morphology. Specifically, GVL/NaOH-LP exhibited a maximum mass of 0.38 ± 0.02 g, representing 14 wt% of the initial raw material. Meanwhile, it showed the lowest purity (47.79% ± 1.44%) and displayed a dispersed morphology with numerous surface pores (Fig. 3A and B). NaOH-LP achieved a mass of 0.12 ± 0.01 g (5 wt% of the initial raw material) and the highest purity of 89.49% ± 4.87%, accompanied by obvious particle agglomeration (Fig. 3A and C). For NaOH/GVL-LP, both the mass of 0.19 ± 0.002 g (8 wt% of the initial raw material) and purity of 82.48% ± 0.001%, fell between GVL/NaOH-LP and NaOH-LP. Similar results were obtained by Sharma *et al.*,^26^ who stated that the purity of extracted lignin determined by the Klason lignin method was 90.86%. Meanwhile, lignin purity varied from 77.5% to 98.5% when moso bamboo was treated by the ternary deep eutectic solvent.^27^ Therefore, the GVL addition affects the efficiency of lignin recovery. Considering the requirement for MB adsorption, GVL/NaOH-LP was chosen to further investigate its adsorption capacity.

### 3.3 Adsorption performance of lignin particles for methylene blue

#### 3.3.1 Adsorption performance

**Fig. 4:**
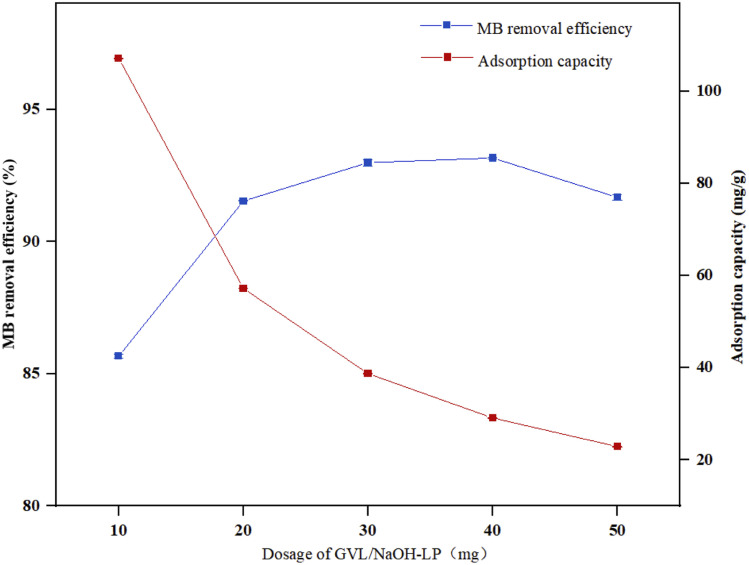
Effect of GVL/NaOH-LP on MB removal efficiency and adsorption capacity.

The MB removal efficiency increased from 85.73% to 91.56% as the dosage of GVL/NaOH-LP rose from 10 to 20 mg (Fig. 4), which is mainly because the additional adsorption sites provided by the increased lignin particle dosage serve as the key factor improving MB removal efficiency under the condition of sufficient MB molecules.^28^ Further increasing the dosage to 40–50 mg, the MB removal efficiency varied from 92.56% to 93.56%, indicating equilibrium in removal performance and full surface saturation.^29^ Correspondingly, the adsorption capacity of GVL/NaOH-LP for MB exhibited a consistent decreasing trend with the increase in lignin particles dosage (Fig. 4). Specifically, as the lignin particles dosage increased from 10 to 50 mg, the adsorption capacity decreased from 107.09 to 22.92 mg g^−1^, representing a 79% reduction. This inverse trend is attributed to particle aggregation and limited MB concentration in solution. Similar findings have been reported by Ogunsile *et al.*,^30^ it demonstrated that the adsorption capacity of lignin particle for target pollutants decreases with an increase in lignin particles dosage. In comparison, conventional alkali-extracted sugarcane bagasse lignin only achieved an adsorption capacity of 9.09 mg g^−1^ under similar test conditions.^31^ The lignin prepared in this study exhibited far superior adsorption performance, which verifies that the synergistic treatment with GVL and NaOH can markedly enhance the adsorption capacity of lignin.

#### 3.3.2 Adsorption kinetics

**Fig. 5:**
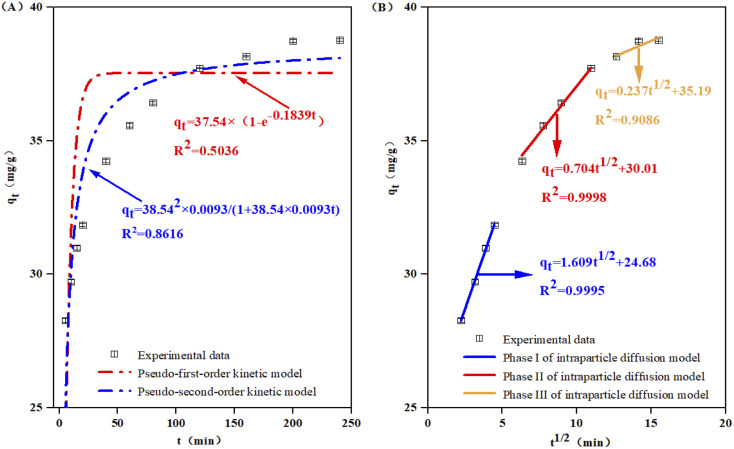
Fitted kinetic curves for MB adsorption on GVL/NaOH-LP. (A) Fitted curves of the pseudo-first- and pseudo-second-order kinetic models; (B) intraparticle diffusion model.

The pseudo-first-order and pseudo-second-order kinetic curves for MB adsorption on GVL/NaOH-LP are shown in Fig. 5A, with the corresponding parameters summarized in Table 3. Compared with the pseudo-first-order kinetic model (*R*_1_^2^ = 0.5036), the pseudo-second-order kinetic model exhibited a superior fitting effect (*R*_2_^2^ = 0.8616), indicating that MB adsorption on GVL/NaOH-LP is dominated by broad-sense chemisorption (mainly hydrogen bonding and weak coordination),while electrostatic interaction and physisorption act as minor contributors. Additionally, the calculated adsorption capacity of the pseudo-second-order model (*q*_e_ = 38.54 mg g^−1^) was in good agreement with the experimentally measured adsorption capacity (*q*_e_ = 38.75 mg g^−1^; Fig. 5A, Table 3). This agrees with previous studies on lignin-based hydrogels, where MB adsorption onto lignin complied with the pseudo-second-order model and achieved MB removal efficiency of 88%.^32^ For MB adsorption, the *q*_*t*_ firstly increased rapidly and then gradually decelerated, finally reaching a plateau within 120 min, indicating that the adsorption equilibrium was achieved (Fig. 5A).

**Table 3:** Kinetic characteristics of MB adsorption on GVL/NaOH-LP

Model		Parameters		
Pseudo-first-order kinetic model		*q*_e1_（mg g^−1^）	*k*_1_	*R*_1_^2^
		37.54	0.1839	0.5036
Pseudo-second-order kinetic model		*q*_e2_（mg g^−1^）	*k*_2_	*R*_2_^2^
		38.54	0.0093	0.8616
Intraparticle diffusion model	First stage	*k*_id1_	*C*_1_	*R*_1_^2^
		1.609	24.68	0.9995
	Second stage	*k*_id2_	*C*_2_	*R*_2_^2^
		0.704	30.01	0.9998
	Third stage	*k*_id3_	*C*_3_	*R*_3_^2^
		0.237	35.19	0.9086

The intraparticle diffusion model results revealed that GVL/NaOH-LP exhibits a multi-stage MB adsorption process. A gradually decreasing adsorption rate was observed in each process, and this variation is primarily attributed to the diffusion of MB from the outer surface to the interior of the GVL/NaOH-LP.^33^ The coefficients of determination for the three stages are *R*_1_^2^ = 0.9995, *R*_2_^2^ = 0.9998, and *R*_3_^2^ = 0.9086 (Fig. 5B, Table 3), indicating an excellent overall goodness of fit. This confirms that intraparticle diffusion acts as a key rate-limiting step in the MB adsorption process of GVL/NaOH-LP. The diffusion rate constants follow the order *k*_id1_ > *k*_id2_ > *k*_id3_, which aligns with the rate variation trend of the three stages described above. Specifically, the largest *k*_id1_ indicates that the surface adsorption proceeds smoothly without obstruction in the first stage. Meanwhile for the second stage, a decreased *k*_id2_ reflects that internal diffusion is hindered by pore resistance. Moreover, the smallest *k*_id3_ confirms that the adsorption rate reaches its minimum after the saturation of adsorption sites in the third stage. The boundary layer thickness parameters are *C*_1_ = 24.68, *C*_2_ = 30.01, and *C*_3_ = 35.19, showing a gradual increasing trend. In the context of intraparticle diffusion modeling, the progressive increase in *C* values across the three stages reflects the sequential process of MB molecules: initial progressive accumulation on the outer surface, followed by their inward diffusion into the interior, which collectively promotes the improvement of MB adsorption capacity on the boundary layer.

#### 3.3.3 Adsorption isotherms

**Fig. 6:**
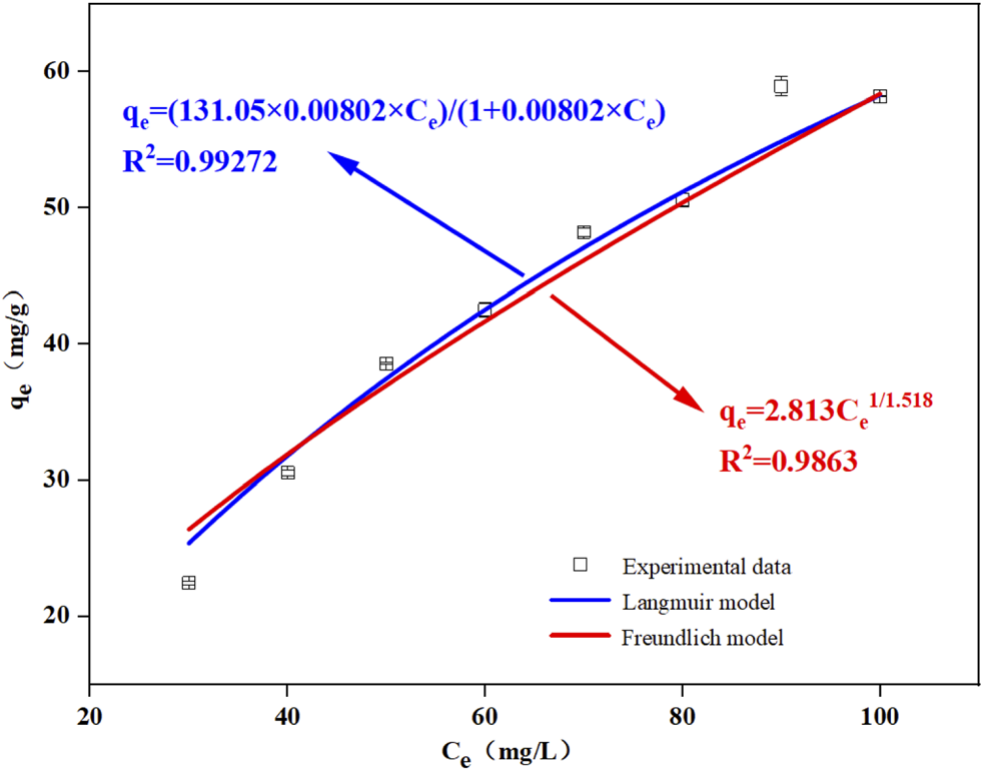
Fitted isotherms for MB adsorption on GVL/NaOH-LP.

The fitted Langmuir and Freundlich isotherm models for MB adsorption on GVL/NaOH-LP are presented in Fig. 6, with the corresponding model parameters summarized in Table 4. For the Freundlich model, the fitting results yielded a coefficient of determination *R*^2^ = 0.9863, along with model parameters *k*_F_ = 2.813 and *n* = 1.518, suggesting a certain degree of heterogeneity in the adsorption process. While, the Langmuir model achieved a higher coefficient of determination (*R*^2^ = 0.9927) compared with the Freundlich model, indicating that the Langmuir model offers better fitting performance in characterizing the adsorption behavior of MB on GVL/NaOH-LP.^34^ The saturated adsorption capacity (*q*_*m*_) derived from the Langmuir model is 131.05 mg g^−1^, confirming that monolayer adsorption is the dominant mechanism for MB adsorption on GVL/NaOH-LP.^35^

**Table 4:** Characteristics of adsorption isotherms for MB adsorption on GVL/NaOH-LP

Model	Parameters			
Freundlich model	*k*_F_		*n*	*R* ^2^
	2.813		1.518	0.9863
Langmuir model	*k*_L_	*q*_m_	*R*_L_	*R* ^2^
	0.00802	131.05	0.55–0.81	0.99272

### 3.4 Mechanism of MB adsorption on lignin particles

Further studies on the MB adsorption mechanism of GVL/NaOH-LP were performed *via* analyzing elemental components, functional groups and mesoporous structure. Regarding elemental composition of GVL/NaOH-LP, the C and H contents are 55.78% and 6.81%, respectively (Table 5). Correspondingly, a H : C ratio of 0.122 was obtained, which confirms the aromatic nature of lignin.^36^ These aromatic sites facilitate π–π stacking interactions with MB molecules, consistent with the observed selectivity in chemisorption kinetics and monolayer adsorption behavior in isotherm models.^37^ Meanwhile, the O : C and (O + N) : C ratios are 0.495 and 0.671, respectively, lower than the typical ranges reported for lignin (O : C 0.6–0.8), indicating reduced polarity and enhanced hydrophobicity for GVL/NaOH-LP.

**Table 5:** Elemental contents, surface area, and pore size of GVL/NaOH-LP

	C (%)	H (%)	O (%)	N (%)	O/C	(O + N)/C	H/C
Element content	55.78	6.81	27.63	9.78	0.495	0.671	0.122
Surface area and pore size	Total surface area (m^2^/g)	Micropore surface area (m^2^/g)	Mesopore surface area (m^2^/g)	Micropore diameter (nm)	Micropore volume (cm^3^ g^−1^)	Mesopore volume (cm^3^ g^−1^)	
	3.856	0.000	3.856	1.002	0.001	0.013	

**Fig. 7:**
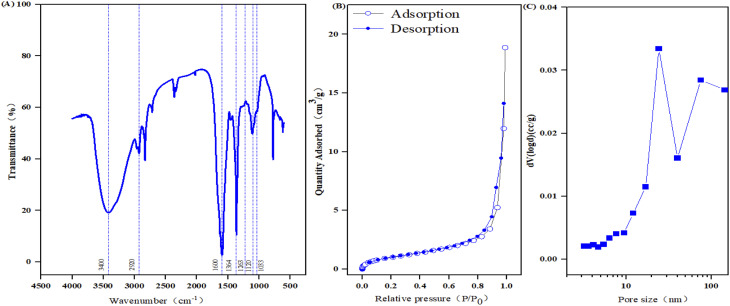
FTIR spectrum and adsorption–desorption isotherm on GVL/NaOH-LP. (A) FTIR spectrum; (B) adsorption–desorption isotherm; (C) pore size distribution.

Meanwhile, the FTIR spectrum of GVL/NaOH-LP exhibits the typical characteristic peaks of lignin (Fig. 7A), with absorption bands observed at 3400 cm^−1^, 2920 cm^−1^, and 1600 cm^−1^. The phenolic hydroxyl groups at 3400 cm^−1^ were assigned to phenolic and aliphatic O–H stretching, providing key structural evidence that chemisorption dominates the pseudo-second-order kinetic model. Subsequently, the band at 2920 cm^−1^ is associated with aliphatic C–H stretching vibrations of methylene (–CH_2_–) groups in lignin. The peak at 1600 cm^−1^ originates from the aromatic C=C stretching vibrations, indicating that the aromatic ring skeleton of lignin is preserved; this is consistent with the high aromaticity inferred from the elemental analysis results. Notably, the absorption peak corresponding to the in-plane bending vibration of aromatic C–H bonds in the syringyl (S) unit of lignin appears at 1136 cm^−1^, which shifts to a lower wavenumber (1120 cm^−1^). This shift suggests that an increased relative content of the S unit may increase the electron density of the aromatic ring, thereby strengthening π–π stacking interactions between lignin and the aromatic moiety of MB. Additionally, the absorption peak at 1364 cm^−1^ is the characteristic peak of hydroxyphenyl groups and *p*-hydroxyphenyl (H) unit of lignin. The absorption peaks at 1263 cm^−1^ and 1033 cm^−1^ (attributed to the guaiacyl (G) and S units, respectively) demonstrate that G and S units are retained in the lignin particles. Alongside the *p*-hydroxyphenyl (H) unit, these structural units collectively ensure the structural stability of the lignin particles and the integrity of their adsorption-active sites, thereby contributing to the favorable adsorption efficiency of the material. All these structural units collectively ensure the structural stability of GVL/NaOH-LP and the integrity of their adsorption-active sites, thereby contributing to the favorable adsorption efficiency of the material.^38^

According to the International Union of Pure and Applied Chemistry (IUPAC) classification, the adsorption–desorption isotherm of GVL/NaOH-LP belongs to type IV curves, accompanied by a distinct H_4_-type hysteresis loop (Figure.7B). Further analysis of the pore size distribution, the most probable pore diameters of GVL/NaOH-LP are 24.1 and 75.4 nm, which fall within the IUPAC-defined mesopore (2–50 nm) and macropore (>50 nm) ranges, respectively (Fig. 7C and Table 5). Furthermore, the surface area of GVL/NaOH-LP is 3.856 m^2^ g^−1^. Regarding pore volume characteristics, the micropores of GVL/NaOH-LP exhibit an average diameter of 1.002 nm and a pore volume of 0.001 cm^3^ g^−1^, whereas the mesopore volume reaches 0.013 cm^3^ g^−1^, which is significantly higher than that of the micropores. These abundant mesopores improve molecular accessibility to surface active sites and accelerate the adsorption kinetics.

In summary, the adsorption of MB on GVL/NaOH-LP is governed by the synergistic effect of π–π stacking interactions, functional groups, and mesopore-facilitated adsorption process.

## 4. Conclusions

This study utilized sweet potato vines as raw material to systematically explore the performance of lignin recovery and its adsorption properties *via* the GVL-mediated route. When GVL was added during the pretreatment phase (Process I, GVL/NaOH-LP), the recovered lignin yield reached 0.38 ± 0.02 g with the lowest purity (47.79% ± 1.44%). For Process III (NaOH/GVL-LP), where GVL was added before acid precipitation, lignin purity reached 82.48% ± 0.001%, alongside a reduced yield of 0.19 ± 0.002 g. Therefore, GVL addition serves as a key factor determining the performance of lignin recovery. MB adsorption on GVL/NaOH-LP agreed well with the pseudo-second-order kinetic. Given the corresponding determination coefficient of 0.8616, the process is mainly controlled by chemisorption, with a maximum adsorption capacity of 38.75 mg g^−1^. Characterizations (SEM, BET, FTIR, elemental analysis) revealed a synergistic adsorption mechanism involving π–π stacking interactions, functional groups, and mesopore-facilitated adsorption process.

## Conflicts of interest

There are no conflicts to declare.

## Data Availability

The authors confirm that all experimental data supporting the findings of this study are available within the article. No additional raw data, software or code are required to understand and reproduce the results.
